# Sonographic estimation of monosodium urate burden predicts the fulfillment of the 2016 remission criteria for gout: a 12-month study

**DOI:** 10.1186/s13075-021-02568-x

**Published:** 2021-07-09

**Authors:** Edoardo Cipolletta, Jacopo Di Battista, Marco Di Carlo, Andrea Di Matteo, Fausto Salaffi, Walter Grassi, Emilio Filippucci

**Affiliations:** Rheumatology Unit, Department of Clinical and Molecular Sciences, Polytechnic University of Marche, “Carlo Urbani” Hospital, Via Aldo Moro 25, Jesi (Ancona), Italy

**Keywords:** Gout, Uric acid, Ultrasound, Remission, Monosodium Urate

## Abstract

**Objective:**

To investigate whether baseline monosodium urate (MSU) burden estimated by ultrasound (US) predicts the achievement of the 2016 remission criteria for gout after 12 months.

**Methods:**

In this 12-month prospective, observational and single-center study, patients with gout fulfilling all the domains of the 2016 preliminary remission criteria for gout at baseline and on urate-lowering therapy (ULT) for at least the preceding 6 months were consecutively enrolled.

The US findings indicative of MSU deposits [aggregates, double contour (DC) sign, and/or tophi] were identified according to the Outcome Measure in Rheumatology US Working Group definitions.

The US MSU burden was estimated by evaluating elbows, wrists, 2nd metacarpophalangeal joints, knees, ankles, and 1st metatarsophalangeal joints.

**Results:**

Remission criteria were fulfilled in 21 (42.0%) out of 50 patients at 12 months. The baseline US MSU burden was significantly lower in patients who achieved remission than in those who did not fulfill the remission criteria at 12 months (1.9±1.8 vs 5.1±3.1, p<0.01).

US scores and ongoing flare prophylaxis were the only significant predictors of remission with an odds ratio of 10.83 [(95%CI=1.14–102.59), p=0.04] for the absence of MSU deposits, 5.53 [(95%CI=1.34–22.76), p<0.01] for the absence of aggregates, 7.33 [(95%CI=1.71–31.44), p<0.01] for the absence of DC sign, 3.88 [(95%CI=1.08–13.92), p=0.04] for the absence of tophi, and 0.23 [(95%CI=0.07–0.75), p=0.02] for ongoing flare prophylaxis.

**Conclusion:**

In gout, baseline US estimation of MSU burden is an independent predictor of the achievement of the remission criteria at 12 months.

**Supplementary Information:**

The online version contains supplementary material available at 10.1186/s13075-021-02568-x.

## Introduction

Gout is characterized by the deposition of monosodium urate (MSU) crystals in joints and periarticular tissues as consequence of a persistent increase of serum urate (SU) above its saturation point [1, 2].

The optimal management of gout is based on three principles: (i) long-term use of urate-lowering therapies (ULT) aiming to achieve target serum urate (SU) levels, (ii) anti-inflammatory treatments for gout flares, and (iii) prophylaxis for gout flares especially in the period shortly after initiating ULT [1, 3–5].

Long-term urate-lowering therapy (ULT) can lead to dissolution of MSU deposits and, consequently, to regression of tophi and prevention of gout flares [3–6].

In 2016, a group of experts developed a set of preliminary remission criteria for gout including the following five domains: SU levels, subcutaneous tophi, gout flares, pain due to gout, and patient global assessment for disease activity, all of which should be measured at least twice over a 12-month interval [7]. In this context, the detection of biomarkers predicting the fulfillment of such criteria may help both clinicians and researchers in refining patients’ stratification.

The construct validity of these criteria has been tested in a recent study using dual-energy computed tomography (DECT) and the prevalence of MSU crystal deposits assessed by DECT resulted significantly lower in patients fulfilling the preliminary remission criteria, compared with patients not fulfilling them [6].

In the last two decades, ultrasonography (US) has emerged as one of the first-choice imaging techniques in the assessment of gout [2, 8, 9]. In fact, there is a consistent body of evidence supporting the validity of US in the detection of MSU deposits [10, 11]. Such evidence led to the inclusion of US in the 2015 American College of Rheumatology (ACR)/European League Against Rheumatism (EULAR) classification criteria and in 2018 updated EULAR evidence-based recommendations for the diagnosis of gout [2, 9]. In addition, several recent studies highlighted the role of US in the management of ULT in gouty patients to monitor the MSU crystals’ dissolution and to decide when stopping gout flare prophylaxis [12–15].

Nevertheless, the role of US in the definition of gout remission has not been explored to date. Therefore, the aim of the present study was to investigate whether baseline MSU burden estimated by US predicts the achievement of remission according to the 2016 preliminary remission criteria for gout.

## Materials and methods

### Study design and patients

In this 12-month prospective, observational and single-center study, patients with gout according to the 2015 ACR/EULAR criteria [2] and on ULT for at least the preceding 6 months were consecutively recruited from the inpatient and outpatient clinics of the Rheumatology Unit of the Polytechnic University of Marche (Italy) from April 2019 to October 2020. Exclusion criteria were: age <18 years old, presence of other inflammatory arthritis, symptomatic peripheral osteoarthritis, or coexisting calcium pyrophosphate deposition disease.

After the baseline clinical assessment, patients without one or more of the following criteria: (1) SU levels <360 μmol/l, (2) absence of subcutaneous tophi, (3) absence of gout flares in the previous month, (4) 0–10-cm pain (due to gout) visual analogue scale (VAS)<2 in the previous month, and (5) 0–10-cm patient global assessment of disease activity VAS<2 in the previous month, were excluded from the subsequent analyses, since they cannot obtain the remission in 1 year (length of the follow-up period specified in the study protocol), defined with at least two measurements of such domains at equal distances apart over 12 months [7]. On the other hand, if patients fulfilled cross-sectionally all of these domains, they were included in the prospective part of the study, since they were potentially able to fulfill the remission criteria over 12 months.

Written informed consent was obtained from all patients before study enrolment for the anonymous collection and analysis of the data. The study was conducted in accordance with Helsinki Declaration and the STrengthening the Reporting of OBservational studies in Epidemiology (STROBE) statement and was approved by the local Ethics Committee (*Comitato Etico Regione Marche -* id CERM: 168/2018).

### Clinical and laboratory evaluations

All patients were assessed by a rheumatologist (J.D.B.) blinded to US findings. Visits were scheduled at 6-month intervals (baseline, 6-month and 12-month visits). During the study period, gout treatment was managed according to the EULAR recommendations [3].

The following data were recorded at baseline: age, gender, body mass index, gout history (i.e., disease duration since the diagnosis of gout, number of flares in the previous 12 months and familiar history of gout), comorbidities, current and previous medications (i.e., ULT, colchicine, steroids and non-steroidal anti-inflammatory drugs), number of subcutaneous tophi, previous results of synovial fluid analyses, C-reactive protein, and current and highest SU levels. As recommended by the Outcome Measure in Rheumatology (OMERACT) Gout Working Group, 0–10-cm pain VAS and 0–10-cm patient global assessment of disease activity VAS with 10 being the worst, and SU levels were registered both at baseline and follow-up visits [7, 16, 17]. In addition, during follow-up visits, patients were asked to report any gout flares, defined as the presence of at least 3 or more of the following criteria: patient-defined flare, pain at rest score higher than 3 on a 0–10 numeric rating scale, presence of at least one swollen joint, and presence of at least one warm joint [18]. Flares were treated according to the EULAR recommendations [3].

In the present study, patients were evaluated three times in 12 months to assess the fulfillment of the provisional remission criteria. In fact, remission was defined as the fulfillment of all following criteria at least twice during 12 months: (1) SU levels <360 μmol/l, (2) absence of subcutaneous tophi, (3) absence of gout flares, (4) 0–10-cm pain (due to gout) VAS<2, (5) 0–10-cm patient global assessment of disease activity VAS<2 [7]. Disease states of gout were defined according to the endorsed Gout, Hyperuricaemia and Crystal-Associated Disease Network (G-CAN) labels and definitions [19].

### Ultrasound assessment

The US examinations were carried out with a Logiq 9 US system (General Electric Medical Systems, USA), working with a linear probe operating at 8–15 MHz. US was performed at baseline by a rheumatologist (E.C.) with 5 years of experience in musculoskeletal US blinded to clinical and laboratory data, who received a specific US training in the field of crystal arthropathies [20]. The US scanning protocol included the bilateral examination of the following anatomic sites: elbow, wrist, 2nd metacarpophalangeal joint, knee, ankle, and 1st metatarsophalangeal joint (Table 1) [21, 22].

**Table 1:** US scanning protocol

Anatomic site	Anatomic target	US findings
Elbow	Triceps tendon	A, T
Wrist	Radiocarpal and intercarpal joints	A, DC, T
Hand	2nd MCPj	A, DC, T
Knee	Patellar tendon	A, T
	HC of the femur	DC
	Popliteal groove region	A, T
Ankle	Achilles tendon	A, T
	Tibiotalar joint	DC
Foot	1st MTPj	A, DC, T

*A* aggregates, *DC* double contour, *HC* hyaline cartilage, *MCPj* metacarpophalangeal joint, *MTPj* metatarsophalangeal joint, *T* tophus, *US* ultrasound

**Fig. 1:**
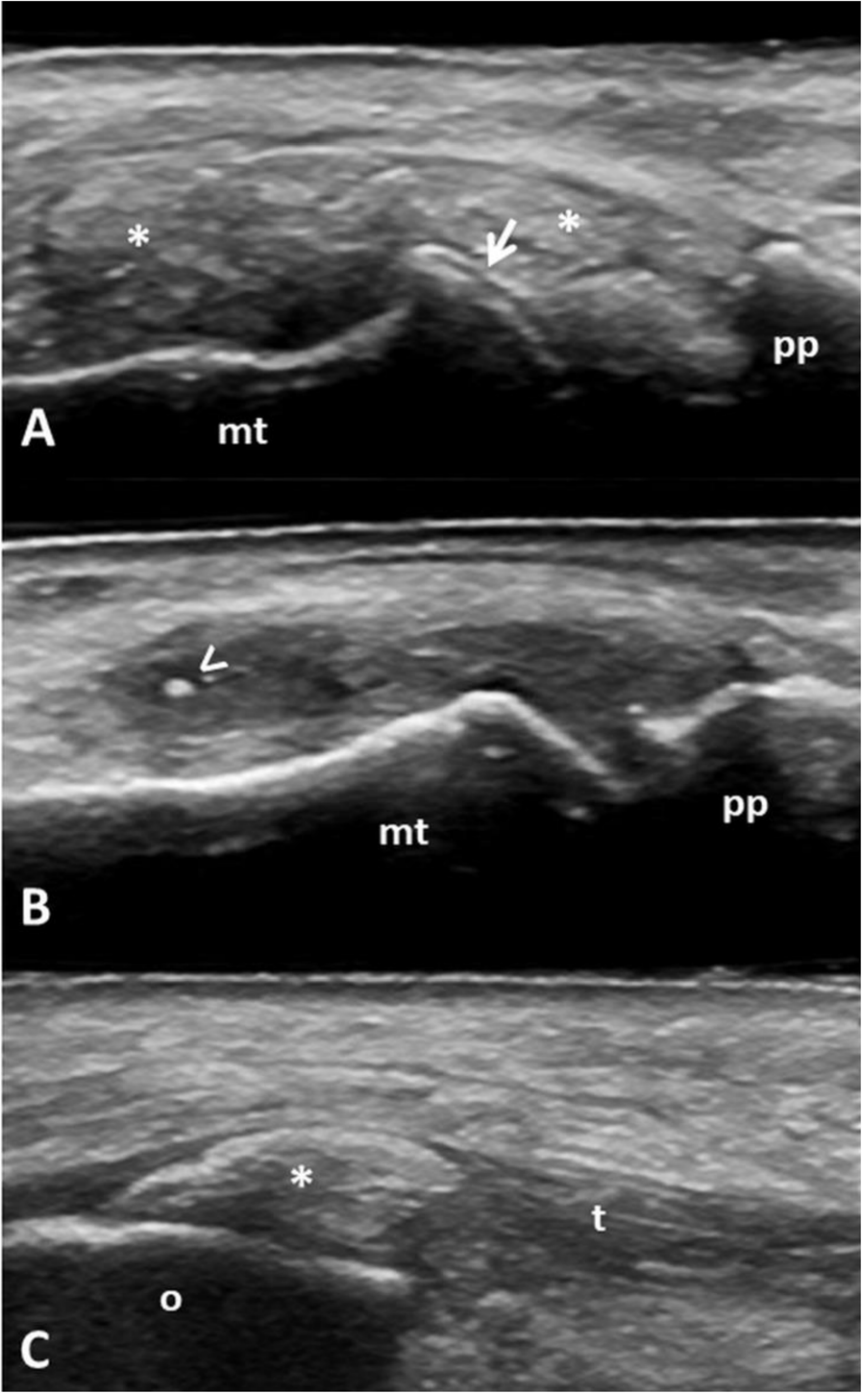
**A** Dorsal longitudinal scan of the 1st metatarsophalangeal joint showing a large “soft” tophus (asterisks) without posterior acoustic shadowing and a double contour sign (arrow) of the metatarsal head cartilage. **B** Dorsal longitudinal scan of the 1st metatarsophalangeal joint revealing a small aggregate (arrowhead). **C** Longitudinal scan of the distal insertion of the triceps tendon into the olecranon process. Note the presence of an intra-tendinous tophus (asterisk). Legend: arrow—double contour sign, arrowhead—aggregate, asterisks—tophi. mt, metatarsal bone; o, olecranon; pp, proximal phalanx; t, triceps tendon

A dynamic and multiplanar examination of each anatomic site was carried out to maximize the identification of MSU deposits [23]. US MSU deposits [aggregates, double contour (DC) sign and tophi] were evaluated according to the OMERACT definitions [24] using a binary score. Gray-scale setting parameters were adapted in order to enhance crystals’ recognition [25, 26]. Figure 1 shows representative examples of US MSU deposits.

Sum scores of aggregates (range 0–14), DC signs (range 0–10), and tophi (range 0–14) were calculated separately, and a total score resulting from the sum of all the elementary US findings (range 0–38) was recorded. The average time required to carry out the US examinations was registered.

### Reliability

The inter- and intra-reader reliability of the sonographer (E.C.) was tested prior to the start of the study, using an expert sonographer (E.F.) as reference standard. Another rheumatologist (J.D.B.) collected a set of 45 US images with MSU deposits from patients with gout and 35 US images without MSU deposits from patients with calcium pyrophosphate deposition disease, osteoarthritis, or rheumatoid arthritis. Images were representative of the whole spectrum of MSU deposits (16 images with DC sign, 15 images with tophi and 14 images with aggregates) in all the anatomical areas examined in the scanning protocol. The two sonographers assessed the images for the presence/absence of MSU deposits to test the inter-reader reliability. The same set of images was evaluated after 2 weeks to test the intra-reader agreement.

### Statistical analysis

Results were reported as mean±standard deviation (SD) for quantitative variables and as frequency and/or corresponding percentage for qualitative variables.

Differences between patients fulfilling or not fulfilling the remission criteria were analyzed using chi-square and Mann-Whitney U tests.

Receiver operating characteristics (ROC) analysis was used to identify cut-off values for each US score which yielded to the highest positive likelihood ratio (LR) and specificity in the identification of patients with the greatest chance to fulfill the remission criteria at 12 months.

Univariate and multivariate logistic regression analyses were carried out to evaluate the predictive values of baseline clinical, laboratory and US findings (independent variables) for the fulfillment of the 2016 preliminary remission criteria at 12 months (dependent variable). All multivariate regression analyses were adjusted for baseline data with a p<0.10 in univariate models. Odds ratio (OR) values were reported with their 95% confidence intervals (95%CI). An OR greater than 1 describes a positive relationship between the independent and the dependent variables, whereas an OR smaller than 1 implies a negative association.

Assuming a prevalence of MSU deposits in patients fulfilling and not fulfilling the remission criteria of 40% and 80%, respectively [6], a study power of 80%, an alpha error of 5%, and an enrolment ratio of 1.5, 47 patients would be needed: 19 in the remission group and 28 in the non-remission group.

Cases with missing data and losses to follow-up were excluded from the analyses. P value lower than 0.05 was considered significant. Statistical analysis was performed using SPSS software (v.26).

## Results

### Study participants and baseline clinical characteristics

Seventy patients with gout fulfilling the 2015 ACR/EULAR classification criteria were consecutively enrolled. Eleven (15.7%) out of 70 patients had a tophaceous gout.

Twenty (28.6%) out of 70 patients were excluded from subsequent analyses. In fact, 13 (18.6%) had SU levels above 360 μmol/l, 11 (15.7%) clinical evidence of subcutaneous tophi, 7 (10.0%) a pain (due to gout) VAS≥2, and 5 (7.1%) a patient global assessment of disease activity VAS≥2. Thus, only 50 (71.4%) out of 70 patients were included in the prospective study. Of them, 48 (96.0%) patients had a crystal-proven diagnosis of gout.

The main baseline demographic, clinical, and laboratory characteristics of the 50 included patients are reported in Table 2. Of note, no significant difference was found between those fulfilling and not fulfilling the remission criteria at 12 months in all the clinical and laboratory data except for gout flare prophylaxis.

**Table 2:** Baseline characteristics of patients with gout fulfilling and not fulfilling the preliminary remission criteria at 12 months

	All patients (n=50)	Fulfilling the criteria (n=21)	Not fulfilling the criteria (n=29)	P value
Age (years, mean±SD)	59.9±14.8	61.5±3.6	58.8±2.6	0.41
Female/male ratio	1/49	1/20	0/29	0.42
Body mass index (kg/m^2^, mean±SD)	27.0±3.3	27.5±3.4	26.6±2.6	0.41
Familiar history of gout [n (%)]	14 (28.0%)	6 (28.6%)	8 (27.6%)	0.94
Disease duration (years, mean±SD)	6.5±6.6	5.9±5.7	6.8±7.2	0.96
Flares in the 12 months preceding the enrolment (mean±SD)	0.8±1.0	0.6±0.9	0.8±1.0	0.25
Smoking habit—past smoker [n (%)]	20 (40.0%)	10 (47.6%)	10 (34.5%)	1.0
Smoking habit—current smoker [n (%)]	7 (14.0%)	3 (14.3%)	4 (13.8%)	
ULT—allopurinol [n (%)]	34 (68.0%)	14 (66.7%)	20 (69.0%)	0.86
ULT—febuxostat [n (%)]	16 (32.0%)	7 (33.3%)	9 (31.0%)	
Ongoing flare prophylaxis with colchicine [n (%)]	27 (54.0%)	7 (33.3%)	20 (69.0%)	**0.02**
Colchicine daily dose (mg, mean±SD)	0.63±0.31	0.50±0.25	0.68±0.31	0.99
Duration of colchicine prophylaxis (months, mean±SD)	9.9±2.2	9.3±2.5	10.1±2.1	0.45
C-reactive protein level (mg/dl, mean±SD)	0.5±0.5	0.4±0.4	0.6±0.5	0.08
SU, highest level (μmol/l, mean±SD)	511.5±95.2	487.7±119.0	535.3±71.4	0.36
SU, current level (μmol/l, mean±SD)	298.4±53.5	285.5±59.5	300.3±53.5	0.16
Number of comorbidities (mean±SD)	3.0±2.0	3.2±2.1	2.8±1.9	0.50
*Dyslipidemia* [n (%)]	22 (44.0%)	11 (52.4%)	11 (37.9%)	0.31
*Arterial hypertension* [n (%)]	31 (62.0%)	13 (61.9%)	18 (62.1%)	0.99
*Ischemic heart disease/Stroke* [n (%)]	5 (10.0%)	1 (4.8%)	4 (13.8%)	0.29
*Atherosclerosis* [n (%)]	14 (28.0%)	7 (33.3%)	7 (24.1%)	0.48
*Nephrolithiasis* [n (%)]	3 (6.0%)	0	3 (10.3%)	0.13
*CKD (eGFR <60 ml/min/1.73m*^*2*^*)* [n (%)]	8 (16.0%)	3 (14.3%)	5 (17.2%)	0.78

*CKD* chronic kidney disease, *eGFR* estimated glomerular filtration rate, *SD* standard deviation, *SU* serum urate, *ULT* urate-lowering therapy

### Fulfillment of the remission criteria at 12 months

Although all the patients satisfied all the remission criteria at baseline, only 21 (42.0%) fulfilled the remission over 12 months. Of the 29 patients not achieving remission at 12 months, 8 (27.6%) did not fulfilled the SU remission domain, 17 (58.6%) the flare domain, 13 (44.8%) the pain domain, and 15 (51.7%) the patient global assessment of disease activity domain.

### Ultrasound findings

MSU deposits were found in at least one scanned site in 14 (66.7%) out of 21 patients fulfilling the remission criteria at 12 months compared with 28 (96.5%) out of 29 patients not fulfilling the remission criteria (p<0.01) (Table 3).

**Table 3:** Prevalence of MSU crystal deposits at baseline in patients fulfilling and not fulfilling the preliminary remission criteria at 12 months

	Patients fulfilling the criteria with US evidence of MSU deposits	Patients not fulfilling the criteria with US evidence of MSU deposits	P value
MSU crystal deposits	14 (66.7%)	28 (96.5%)	**<0.01**
Aggregates	10 (47.6%)	25 (86.2%)	**<0.01**
DC sign	4 (19.0%)	17 (58.6%)	**<0.01**
Tophi	8 (38.1%)	20 (69.0%)	**0.03**

*DC* double contour, *MSU* monosodium urate, *US* ultrasound

Patients fulfilling the preliminary remission criteria (n=21), patients not fulfilling the preliminary remission criteria (n=29)

The absence of US findings indicative of MSU deposits at baseline was significantly correlated with the fulfillment of the remission criteria at 12 months, whereas the US detection of MSU deposits was negatively associated with their fulfillment. In fact, 87.5% of patients without US evidence of MSU crystals in all the scanned sites achieved the remission in comparison with 33.3% with US evidence of MSU deposits in at least one anatomic site (p<0.01). On the other hand, 66.7% of patients with US evidence of MSU deposits in at least one anatomic site did not reach the remission in comparison with 12.5% without US evidence of MSU crystals in all the scanned sites (p<0.01) (Supplementary Figure 1).

Furthermore, patients found in remission at 12 months had a smaller baseline US MSU burden considering each US elementary finding (Table 4 and Supplementary Figure 2).

**Table 4:** US scores of MSU crystal deposits in patients fulfilling and not fulfilling the preliminary remission criteria at 12 months

	All patients (n=50)	Fulfilling the criteria (n=21)	Not fulfilling the criteria (n=29)	P value
Total score (range 0–38)	3.8±3.0	1.9±1.8	5.1±3.1	<0.01
Aggregates score (range 0–14)	1.9±1.7	1.1±1.4	2.5±1.8	<0.01
DC sign score (range 0–10)	0.8±1.1	0.2±0.4	1.2±1.2	<0.01
Tophi score (range 0–14)	1.1±1.3	0.6±0.9	1.5±1.4	0.02

*DC* double contour, *MSU* monosodium urate, *US* ultrasound

Aggregates were most frequently detected at knee level, while DC sign and tophi at 1st metatarsophalangeal joint level. Supplementary Table 1 provides a detailed description of the topographic distribution of US findings indicative of MSU deposits at baseline.

The average time required to complete the US examination was 30±4 min.

Kappa values for the inter- and intra-reader agreement were 0.73 (95%CI 0.54 to 0.93) and 0.77 (95%CI 0.59–0.96).

### Predictive values of baseline data for the fulfillment of the remission criteria at 12 months

US scores and gout flare prophylaxis were the only variables significantly associated with remission in the univariate analyses (Table 5) with an OR of 10.83 [(95%CI = 1.14–102.59), p = 0.04] for the absence of US MSU deposits, 5.53 [(95%CI = 1.34–22.76), p < 0.01] for the absence of aggregates, 7.33 [(95%CI = 1.71–31.44), p < 0.01] for the absence of DC sign score, 3.88 [(95%CI = 1.08–13.92), p = 0.04] for the absence of tophi, and 0.23 [(95%CI = 0.07–0.75), p = 0.02] for the ongoing flare prophylaxis. As the latter variable may have an impact on the achievement of remission, we performed multivariate analyses adjusted for clinical and laboratory confounders. Despite this, US scores remained independent predictors of remission in the multivariate analyses (Table 5).

**Table 5:** Predictive values of baseline data for the fulfillment of the remission criteria at 12 months

	OR (95%CI)	P value
**Univariate analyses**		
Absence of gout flares in the previous 12 months	2.48 (0.82–5.88)	0.95
Age	1.01 (0.97–1.05)	0.52
Body mass index	1.10 (0.91–1.35)	0.33
C reactive protein within normal range	3.0 (0.80–11.19)	0.10
Current SU level	0.61 (0.33–1.15)	0.13
Disease duration	0.98 (0.90–1.07)	0.63
Familiar history of gout	0.95 (0.27–3.32)	0.94
Gender	0.96 (0.75–1.57)	0.74
Highest ever SU level <535 μmol/l	2.60 (0.69–9.75)	0.15
Number of comorbidities	1.12 (0.83–1.49)	0.46
Ongoing flare prophylaxis	0.23 (0.07–0.75)	**0.02**
Smoking status (current or previous smoker)	1.33 (0.24–7.56)	0.60
Absence of MSU crystal deposits—total score=0	10.83 (1.14–102.59)	**0.04**
Absence of aggregates—aggregate score=0	5.53 (1.34–22.76)	**<0.01**
Absence of DC sign—DC sign=0	7.33 (1.71–31.44)	**<0.01**
Absence of tophi—Tophus score=0	3.88 (1.08–13.92)	**0.02**
**Multivariate analyses**		
Absence of MSU crystal deposits—total score=0	10.13 (1.02–100.28)	**0.04**
Absence of aggregates—aggregate score=0	4.80 (1.22–27.92)	**0.03**
Absence of DC sign—DC sign=0	6.61 (1.83–29.67)	**0.01**
Absence of tophi—Tophus score=0	3.80 (1.05–13.75)	**0.04**

*DC* double contour, *MSU* monosodium urate, *OR* odds ratio, *SU* serum urate

Multivariate analyses were adjusted for the following variables: C-reactive protein within normal range and ongoing flare prophylaxis

As shown in Supplementary Table 2, a cut-off value of 0 had the highest LH+ in the identification of those patients with the greatest chance to fulfill the remission criteria at 12 months.

The risk of not fulfilling the remission criteria increased with the US burden of MSU deposits. For each 1-point increase in total score, aggregate score, DC sign score, and tophus score, the risk increased by 1.81- (95%CI 1.27–2.60), 1.73- (95%CI 1.14–2.64), 4.16- (95%CI 1.55–11.3), and 1.95-fold (95%CI 1.07–3.56), respectively. These last analyses were adjusted for the following confounders: ongoing flare prophylaxis and C-reactive protein within normal range.

## Discussion

The aim of treatment in many rheumatic diseases is to reach a state of remission. Since gout clinical manifestations arise from MSU crystals deposition, the main aim of gout management is to achieve their complete disappearance from body tissues after an effective ULT [27–31].

In 2016, a large group of gout-experts published preliminary criteria for gout remission including clinical and laboratory data that need to be fulfilled at least twice over a 12-month interval [7]. Although imaging techniques (e.g., US and DECT) provide a direct visualization of MSU crystals and allow to estimate and monitor the burden of MSU deposits [32], an imaging domain was not included in such criteria.

The results of our study suggest that US MSU burden predicts the fulfillment of the preliminary remission criteria for gout: the lower the baseline MSU burden estimated by US, the higher the chance to fulfill the remission criteria at 12 months. Therefore, US may improve risk-stratification and inform management of these patients. First, the absence of US MSU deposits, that can be considered the closest condition to an ideal definition of gout remission, was the strongest predictor of remission (LR+=11.0 and OR=10.83). In fact, 87.5% of patients without US evidence of MSU deposits, but only 33.3% with US MSU deposits fulfilled the remission criteria at 12 months (p < 0.01). On the other hand, our results indicate that a greater US MSU burden at baseline was associated with a higher risk of not fulfilling remission criteria at 12 months. Among the elementary US findings indicative of MSU deposits, DC sign can be considered the most relevant. In fact, 81.0% of the patients with US evidence of DC sign did not reach the remission at 12 months (Supplementary Figure 1). This is not surprising if we consider that DC sign has been regarded as a key US feature in gout. In fact, it is one of the most common US finding indicating MSU deposits in patients with both early and established gout [11, 33]. Moreover, it was the most sensitive to change US feature in patients undergoing ULT [12–15, 34]. Therefore, the presence of DC sign is a strong imaging biomarker that should induce the clinicians to maintain or even to increase the ULT.

Our results are in line with the data obtained by Dalbeth et al. who found imaging evidence of MSU deposits using DECT in 44% of subjects fulfilling the preliminary remission criteria [6]. In fact, although the US MSU burden was significantly lower in patients who fulfilled the remission criteria, MSU deposits were still detectable by US in a relevant number of these patients ranging from 19.0% with DC sign to 47.6% with aggregates. The US identification of aggregates in patients in clinical remission (47.6%) may be explained by the new formation of aggregates from the dissolution of larger tophi [12, 13] or by the misinterpretation of non-crystalline hyperechoic spots [26], as the specificity of OMERACT-defined aggregates has been recently questioned [35]. The persistence of tophi even in patients in clinical remission (38.1%) may be related to a “dense” collection of MSU crystals requiring a more aggressive or a more prolonged ULT or to the histological composition of the tophus itself. In fact, some studies have hypothesized that the surrounding fibrotic tissue or peri-tophaceous calcific deposits may persist despite an effective ULT [34, 36, 37]. Thus, clinical remission can be recorded at 12 months also in patients with imaging evidence of MSU deposits. However, the percentage of patients in imaging remission, defined as the absence of US MSU deposits, resulted ten times higher in patients in clinical remission than in those not fulfilling the remission criteria (Table 3, 33.3% vs 3.5%, p<0.01).

Whether reaching and maintaining an imaging remission may improve long-term outcomes is still an unexplored issue. However, the US evidence of MSU deposits has been linked to clinical measures of functional impairment and disability in patients with both gout and asymptomatic hyperuricemia even in the absence of clinical evidence of inflammation [38, 39]. In addition, the potential deleterious impact of a persistent MSU crystal load on cardiac and renal outcomes has been reported by several research groups [40–43]. Therefore, it is reasonable to hypothesize that the persistence of subclinical MSU deposits may be associated with worse outcomes and that true remission should include an imaging domain.

In our cohort, only 42.0% of patients amenable to fulfill the remission criteria at baseline achieved the remission at 12 months. This observation supports the need for a longitudinal assessment of gout remission over a 12-month interval. In previous studies, a variable percentage of patients, ranging from 9.1 to 48.2% [6, 44, 45], reached the 2016 remission criteria [7]. This heterogeneity can be explained by several factors: first, the inclusion of patients with different clinical and laboratory characteristics (e.g., presence/absence of subcutaneous tophi and different disease duration); second, the use of different ULTs (e.g., allopurinol, febuxostat and pegloticase); third, the duration of ULT prior to the enrolment; and finally, the length of the observational period (from 12 to 60 months) [5, 43, 44]. Interestingly, Alvarado-de la Barrera and colleagues found that the proportion of patients achieving the remission increased with the duration of ULTs [45]. In fact, 9.1%, 30%, and 39% of patients with a non-severe tophaceous gout (less than 4 subcutaneous tophi) were in remission after 12, 24, and 48 months, respectively [45]. Furthermore, none of those with a severe tophaceous gout (more than 4 subcutaneous tophi) achieved the remission even after 60 months [45].

Another interesting observation is that the fulfillment of the remission criteria was obtained more frequently by patients who did not require prophylaxis for gout flares. This could be explained by the positive association between disease activity, risk of flares, and MSU burden [14, 46–50].

Finally, although allopurinol may have a variety of beneficial effects beyond urate-lowering action [51], in our study a similar proportion of patients treated with allopurinol and with febuxostat achieved the remission (p = 0.86).

The strengths of the present study are the prospective design and the adoption of international and consensus-based definitions of clinical states (i.e., remission and acute flare) and of US MSU deposits [7, 18, 24]. Furthermore, the US scanning protocol included many anatomic targets including both upper and lower limbs and both joints and tendons, allowing to estimate the “whole-body” MSU burden. However, the higher is the number of scanned anatomic sites, the higher is also the time required to carry out the US examination. Nevertheless, when US is performed to estimate the MSU burden once a year, an average time of 30 min to complete the US examination could be considered reasonable.

The limitations of this study include: the single-center design, the lack of a direct comparison between US and DECT findings, and the fact that US examinations were carried out by a single sonographer. However, the inter- and intra-reader reliability was found to be substantial. In addition, MSU deposits were scored using a binary system, and this limits the estimation of MSU burden. However, to date, no consensus exists on a standardized and reproducible method to score MSU deposits either semiquantitatively or quantitatively. Finally, the study design does not allow to weight the value of US findings in the subgroup of patients with high probability of not fulfilling the preliminary remission criteria on the basis of baseline clinical and laboratory data (i.e., with clinical evidence of tophi). Nevertheless, in such a clinical setting, the additional value of US findings could be low in predicting remission.

Further studies should address the accuracy and the feasibility of a reduced scanning protocol for evaluating the burden of MSU crystal deposits and whether a more accurate estimation of MSU burden could be obtained by using either a semiquantitative scoring system or a quantitative method (i.e., the measurement of an index tophus) [52].

## Conclusions

Baseline US estimation of MSU burden is an independent predictor of gout clinical remission at 12 months. The absence of US MSU deposits was the most significant predictor of remission, whereas the US detection of DC sign in at least one joint of a failure in achieving remission. Thus, performing an US examination in patients amenable to fulfill the remission criteria after 12 months may improve risk-stratification and inform management of these patients. Although the results of our study provide further evidence supporting the concurrent validity of the 2016 preliminary remission criteria, it is noteworthy that MSU deposits may still be detectable in a half of patients fulfilling these criteria, posing the question whether there is a need for adding an imaging domain in such criteria to explore the subclinical burden of MSU deposits.

## Supplementary Information

**Additional file 1: Supplementary Table 1.** Topographic distribution of US features indicating MSU crystal deposits in patients fulfilling and not fulfilling the preliminary remission criteria at 12 months. **Supplementary Table 2**. Identification of the optimal cut-off values of US scores. **Supplementary Figure 1.** The figure reports the percentages of patients with and without baseline US evidence of MSU deposits found to fulfill or not the remission criteria at 12 months (total number of patients with baseline US evidence of MSU deposits=42, 25, 21, 28; respectively) (total number of patients without US evidence of MSU deposits=8, 25, 29, 22; respectively). **Supplementary Figure 2.** Percentages of patients fulfilling the remission criteria (blue columns) and not fulfilling the remission criteria (orange columns) at 12 months with respect to the US scores.

## Data Availability

The datasets used and/or analyzed during the current study are available from the corresponding author on reasonable request.

## Abbreviations

95%CI: 95% confidence interval
ACR: American College of Rheumatology
CKD: Chronic kidney disease
DC: Double contour
eGFR: Estimated glomerular filtration rate
EULAR: EUropean League Against Rheumatism
LR: Likelihood ratio
MSU: Monosodium urate
NSAID: Non-steroidal anti-inflammatory drug
OMERACT: Outcome Measure in Rheumatology
OR: Odds ratio
ROC: Receiver operating characteristics
SD: Standard deviation
STROBE: STrengthening the Reporting of OBservational studies in Epidemiology
SU: Serum urate
VAS: Visual analogue scale
ULT: Urate-lowering therapy
US: Ultrasound
