# Supplementary material for: Sonographic estimation of monosodium urate burden predicts the fulfillment of the 2016 remission criteria for gout: a 12-month study
Source: Arthritis Res Ther. 2021 Jul 9;23:185. doi: 10.1186/s13075-021-02568-x (PMC8268270; doi:10.1186/s13075-021-02568-x)
Supplement: Supplementary file 1 — Additional file 1: Supplementary Table 1. Topographic distribution of US features indicating MSU crystal deposits in patients fulfilling and not fulfilling the preliminary remission criteria at 12 months. Supplementary Table 2. Identification of the optimal cut-off values of US scores. Supplementary Figure 1. The figure reports the percentages of patients with and without baseline US evidence of MSU deposits found to fulfill or not the remission criteria at 12 months (total number of patients with baseline US evidence of MSU deposits=42, 25, 21, 28; respectively) (total number of patients without US evidence of MSU deposits=8, 25, 29, 22; respectively). Supplementary Figure 2. Percentages of patients fulfilling the remission criteria (blue columns) and not fulfilling the remission criteria (orange columns) at 12 months with respect to the US scores. [file 13075_2021_2568_MOESM1_ESM.docx]

| **Supplementary Table 1**. Topographic distribution of US features indicating MSU crystal deposits in patients fulfilling and not fulfilling the preliminary remission criteria at 12 months. | | | | | | | | | | | | |
| --- | --- | --- | --- | --- | --- | --- | --- | --- | --- | --- | --- | --- |
| **Anatomic target** | **Aggregates** | | | | **DC sign** | | | | **Tophi** | | | |
|  | **All patients** | **Fulfilling the criteria** | **Not fulfilling the criteria** | **P value** | **All patients** | **Fulfilling the criteria** | **Not fulfilling the criteria** | **P value** | **All patients** | **Fulfilling the criteria** | **Not fulfilling the criteria** | **P value** |
| Triceps tendon | 14 (14.0%) | 2 (4.8%) | 12 (20.7%) | **0.04** | NA | NA | NA | / | 3 (3.0%) | 0 | 3 (5.1%) | 0.26 |
| Wrist | 9 (9.0%) | 1 (2.4%) | 8 (13.8%) | 0.08 | 1 (1.0%) | 0 | 1 (1.7%) | 1.0 | 3 (3.0%) | 0 | 3 (5.1%) | 0.26 |
| 2^nd^ MCPj | 6 (6.0%) | 0 | 6 (10.3%) | **0.04** | 5 (5.0%) | 0 | 5 (8.6%) | 0.07 | 3 (3.0%) | 0 | 3 (5.1%) | 0.26 |
| Distal femur hyaline cartilage | NA | NA | NA | / | 21 (21%) | 1 (2.4%) | 20 (34.5%) | **<0.01** | NA | NA | NA | / |
| Popliteal groove region | 29 (29.0%) | 6 (14.3%) | 23 (39.7%) | **<0.01** | NA | NA | NA | / | 7 (7.0%) | 2 (4.8%) | 5 (8.6%) | 0.70 |
| Patellar tendon | 17 (17.0%) | 3 (7.1%) | 14 (24.1%) | **0.03** | NA | NA | NA | / | 9 (9.0%) | 0 | 9 (15.5%) | **<0.01** |
| Talar hyaline cartilage | NA | NA | NA | / | 16 (16.0%) | 2 (4.8%) | 14 (24.1%) | **0.01** | NA | NA | NA | / |
| Achilles tendon | 12 (12.0%) | 1 (2.4%) | 11 (19.0%) | 0.09 | NA | NA | NA | / | 11 (11.0%) | 1 (2.4%) | 10 (17.2%) | **0.02** |
| 1^st^ MTPj | 24 (24.0%) | 10 (23.8%) | 14 (24.1%) | 1.0 | 15 (15.0%) | 1 (2.4%) | 14 (24.1%) | **<0.01** | 39 (39.0%) | 10 (23.8%) | 29 (50.0%) | **0.01** |
| **DC**: double contour sign, **MCPj**: metacarpophalangeal joint, **MTPj**: metatarsophalangeal joint, **NA**: not assessed.  Percentages were referred to the number of scanned anatomic targets (n=100 in the columns under the heading “All patients”, n=42 in the columns under the heading “Fulfilling the criteria” and n=58 in the columns under the heading “Not fulfilling the criteria”). | | | | | | | | | | | | |

| **Supplementary Table 2.** Identification of the optimal cut-off values of US scores. | | | | | |
| --- | --- | --- | --- | --- | --- |
| **Total score. AUC**: 0.82, **95%CI**: 0.71-0.94, **SE**: 0.06, p**<0.01** | | | | | |
| Cut off value | Sensitivity | Specificity | LR+ | LR- | Youden Index |
| 0 | 0.33 | 0.97 | 11.0 | 0.7 | 0.3 |
| ≤1 | 0.43 | 0.93 | 6.1 | 0.6 | 0.36 |
| ≤2 | 0.71 | 0.79 | 3.4 | 0.4 | 0.50 |
| ≤3 | 0.71 | 0.65 | 2.0 | 0.4 | 0.36 |
| ≤4 | 0.91 | 0.52 | 1.9 | 0.2 | 0.43 |
| ≤5 | 1.0 | 0.41 | 1.7 | 0.0 | 0.41 |
| **Aggregate score. AUC**: 0.73, **95%CI**: 0.59-0.87, **SE**: 0.07, p**<0.01** | | | | | |
| Cut off value | Sensitivity | Specificity | LR+ | LR- | Youden Index |
| 0 | 0.52 | 0.86 | 3.7 | 0.6 | 0.36 |
| ≤1 | 0.62 | 0.65 | 1.8 | 0.6 | 0.27 |
| ≤2 | 0.86 | 0.41 | 1.5 | 0.3 | 0.27 |
| ≤3 | 0.91 | 0.31 | 1.3 | 0.3 | 0.22 |
| ≤4 | 1.0 | 0.17 | 1.2 | 0.0 | 0.17 |
| **DC sign score. AUC**: 0.74, **95%CI**: 0.61-0.88, **SE**: 0.07, p**<0.01** | | | | | |
| Cut off value | Sensitivity | Specificity | LR+ | LR- | Youden Index |
| 0 | 0.81 | 0.59 | 2.0 | 0.3 | 0.40 |
| ≤1 | 1.0 | 0.45 | 1.8 | 0.0 | 0.45 |
| **Tophus** **score. AUC**: 0.68, **95%CI** 0.53-0.83, **SE**: 0.08, p=**0.03** | | | | | |
| Cut off value | Sensitivity | Specificity | LR+ | LR- | Youden Index |
| 0 | 0.62 | 0.69 | 2.0 | 0.6 | 0.31 |
| ≤1 | 0.81 | 0.48 | 1.6 | 0.4 | 0.29 |
| ≤2 | 0.95 | 0.14 | 1.1 | 0.4 | 0.09 |
| ≤3 | 1.0 | 0.07 | 1.1 | 0.0 | 0.07 |
| **95%CI**: 95% confidence of interval; **AUC**: area under the curve, **LR-**: negative likelihood ratio, **LR+**: positive likelihood ratio, **SE**: standard error. | | | | | |

**Supplementary Figure 1.**

**
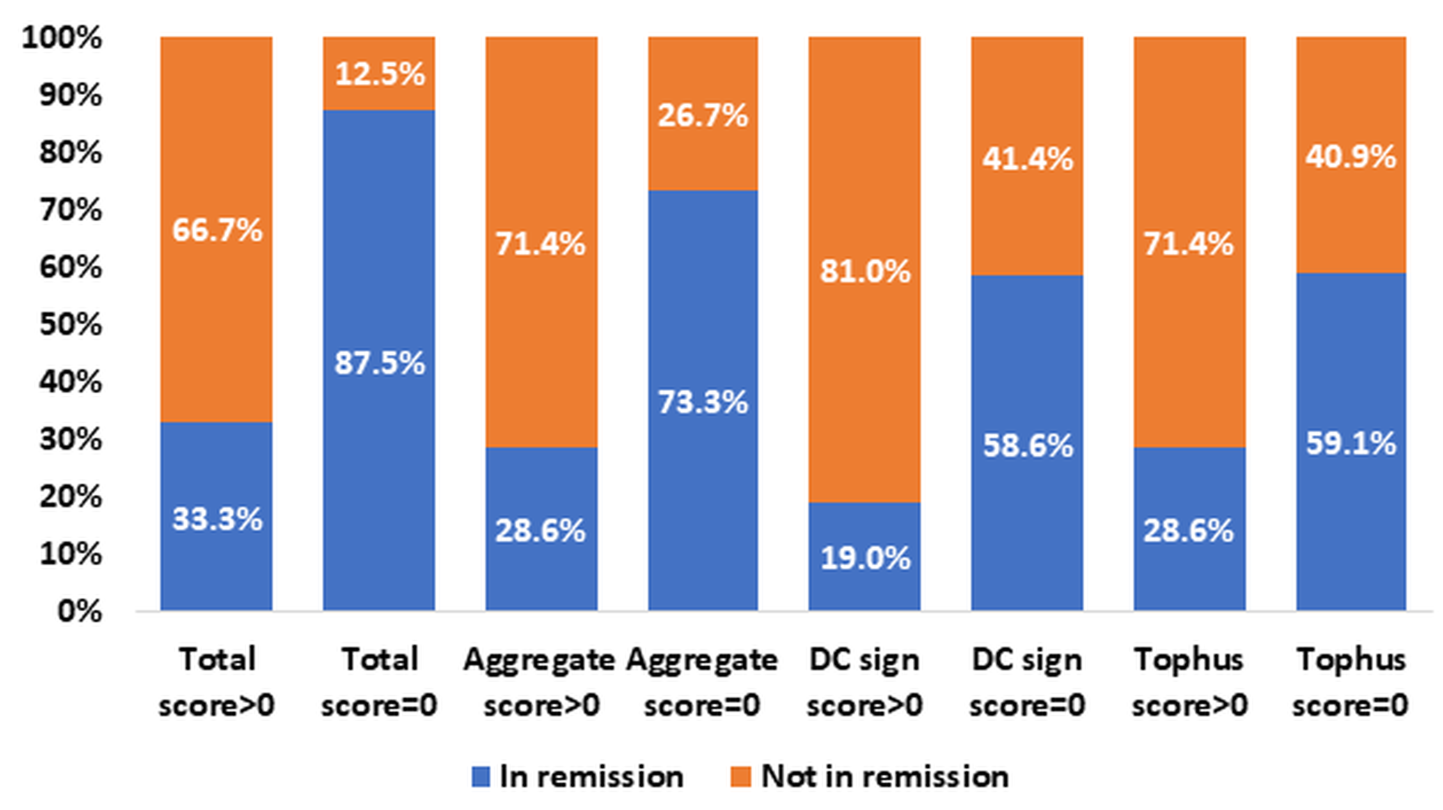
**

The figure reports the percentages of patients with and without baseline US evidence of MSU deposits found to fulfill or not the remission criteria at 12 months (total number of patients with baseline US evidence of MSU deposits=42, 25, 21, 28; respectively) (total number of patients without US evidence of MSU deposits=8, 25, 29, 22; respectively).

**Supplementary Figure 2**.

**
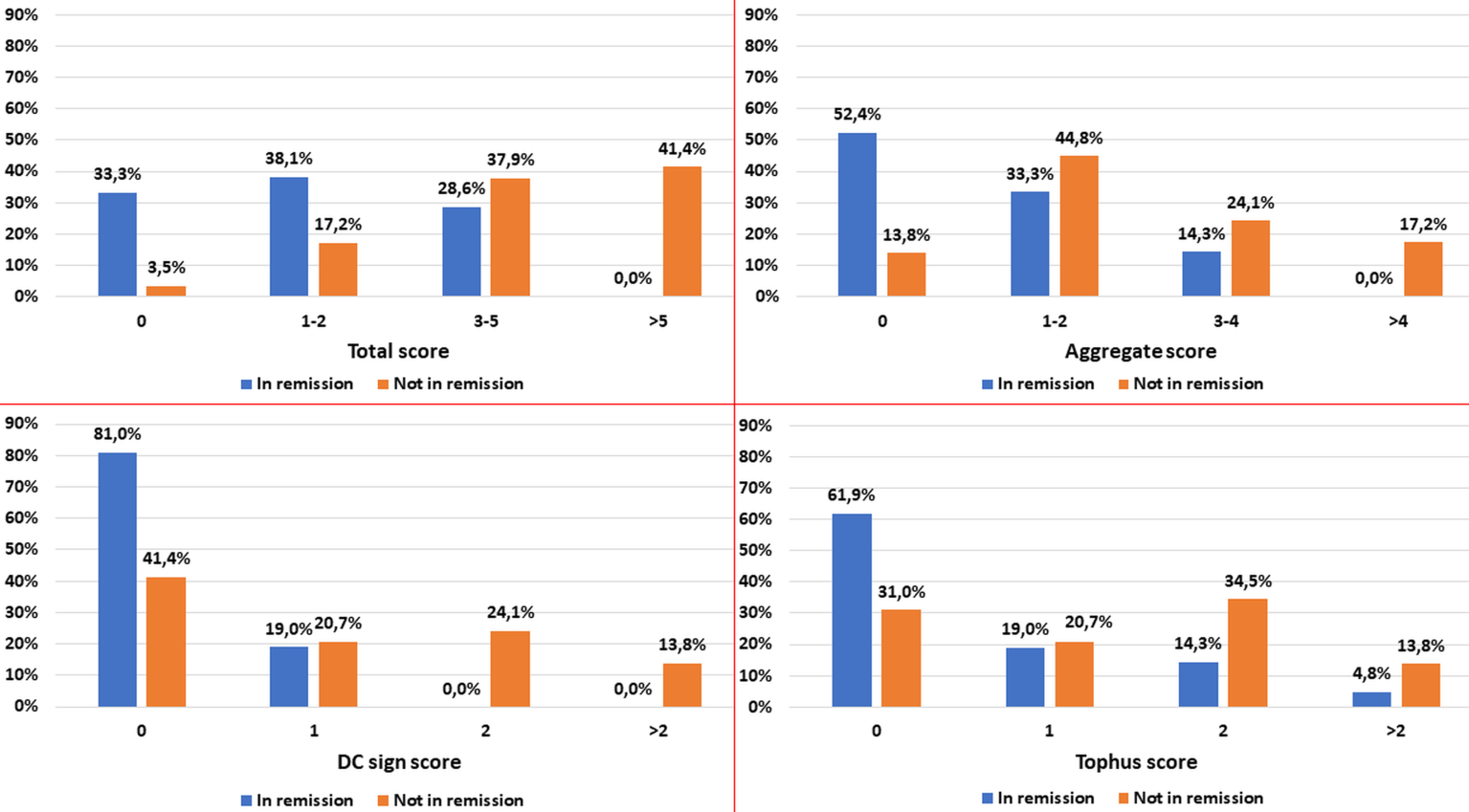
**
